# Astrocyte heterogeneity in the brain: from development to disease

**DOI:** 10.3389/fncel.2015.00076

**Published:** 2015-03-20

**Authors:** Clarissa Schitine, Luciana Nogaroli, Marcos R. Costa, Cecilia Hedin-Pereira

**Affiliations:** ^1^ Cellular Neuroanatomy Laboratory, Program in Neurobiology, Institute of Biophysics Carlos Chagas Filho, Federal University of Rio de Janeiro, Rio de JaneiroBrazil; ^2^ Laboratory of Cellular Neurobiology, Brain Institute, Federal University of Rio Grande do Norte, NatalBrazil; ^3^ Oswaldo Cruz Institute, FIOCRUZ, Rio de JaneiroBrazil

**Keywords:** astrocyte, heterogeneity, progenitors, cerebral cortex, psychiatric diseases

## Abstract

In the last decades, astrocytes have risen from passive supporters of neuronal activity to central players in brain function and cognition. Likewise, the heterogeneity of astrocytes starts to become recognized in contrast to the homogeneous population previously predicted. In this review, we focused on astrocyte heterogeneity in terms of their morphological, protein expression and functional aspects, and debate in a historical perspective the diversity encountered in glial progenitors and how they may reflect mature astrocyte heterogeneity. We discussed data that show that different progenitors may have unsuspected roles in developmental processes. We have approached the functions of astrocyte subpopulations on the onset of psychiatric and neurological diseases.

## Astrocyte Diversity

The human brain is acknowledged as the most complex of all organs, a structure dynamic enough to adapt to dramatic environmental changes through plasticity occurring as a result of normal physiology or during pathological events. Such complexity requires powerful homeostasis processes to assure the perfect functioning of the system (Scuderi et al., 2013). Glial cells are pivotal to maintain the structural integrity and functionality required by the central nervous system (CNS). The so-called macroglia consists of a heterogeneous cell population, which comprises ependymal cells, astrocytes, oligodendrocytes, and NG2 cells. Each of these cell populations is unique, although sharing some properties.

The initial concept of macroglial cells as passive in brain function, working as a supportive element for the maintenance of neurons, has dramatically changed in the last quarter of a century with an increased body of evidence showing active roles of glial cells in the transmission and integration of neural information (Wang and Bordey, 2008). In fact, macroglial cells are directly involved in neuronal function through controlling neurogenesis, synaptogenesis, neurotransmission, synaptic plasticity, neuronal growth, and neuron survival (Scuderi et al., 2013; Zhang and Barres, 2013).

Astrocytes represent the most abundant macroglia and the largest and most heterogeneous group of glial cell types. They participate in a wide variety of complex and essential functions in the brain. For instance, astrocytes are responsible for the reuptake and release of transmitters, distribution of water, pyruvate metabolism, removal of reactive oxygen species, and antioxidant (glutathione) metabolism, organization of the blood brain barrier, ion buffering, synthesis, and secretion of trophic factors and release of gliotransmitters through exocytosis mechanism (Sofroniew and Vinters, 2010; Scuderi et al., 2013).

Albeit the heterogeneity of functions and the prominent roles they exert, astroglial cells have mostly been overlooked in the quest to understand healthy and diseased brain functioning and the classification of astroglial cells still relies greatly on morphological criteria and use of few molecular markers.

Astrocytes are classically divided into two major subpopulations in cerebral cortex: fibrous astrocytes in the white matter and protoplasmic astrocytes in the gray matter (Miller and Raff, 1984). Fibrous astrocytes have long, thin processes, yielding a star-like appearance. Protoplasmic astrocytes have many branching processes, which contact and ensheath synapses, and usually have one or two processes in contact with blood vessels. However, this classification is outdated in light of the great diversity of astrocytes revealed by more detailed morphological and biochemical analyses.

In fact, mature astrocytes can be identified by the expression of glial fibrillary acidic protein (GFAP), calcium-binding protein S100β, glutamate–aspartate transporter and glutamate transporter 1 (GLT-1), and additional markers is recently suggested based on microarray gene expression profiles (Bachoo et al., 2004). Expressions of these markers, as well as astrocyte morphologies, vary considerably amongst cortical regions (Emsley and Macklis, 2006; Regan et al., 2007), suggesting that astrocyte subpopulations could be differentially specified to display distinct biochemical/biophysical properties throughout discrete regions of the cerebral cortex (Emsley and Macklis, 2006).

Astrocyte heterogeneity is also appreciated in other CNS regions. Recent data suggest that the differential expression of ionotropic receptors in thalamic astrocytes could indicate functional heterogeneity. The thalamus, responsible for processing sensory information relayed to the cortex, contains two different astrocyte populations regarding the expression of glutamatergic receptors. Thalamic astrocytes isolated from postnatal transgenic mice expressing human GFAP promoter under the control of enhanced green fluorescent protein (EGFP) were stimulated with kainate, and kainate plus cyclothiazide (CTZ), an AMPA receptor modulator. Only 60% of the cells stimulated showed enhanced inward currents upon kainate and CTZ application (Hoft et al., 2014). In addition, all astrocytes observed in electrophysiological recordings showed K^+^ currents upon muscimol stimulus, a selective agonist for the GABA-A receptor (Hoft et al., 2014). These data indicate that astrocyte subpopulations differentially express neurotransmitter receptors, reflecting a putative difference in astrocytic function and physiology.

Using the same transgenic mice model above, two different populations of astrocytes were identified in hippocampal freshly isolated cells or brain slices (Matthias et al., 2003). One population of cells displays weak GFAP fluorescence, thin and short processes, whereas the second group of cells displays intense GFAP-EGFP labeling and more complex process morphology (Matthias et al., 2003). Besides morphology, the two groups of cells differ in their electric properties. In whole cell recordings, the first group of cells shows an outward rectifying K^+^ current and the second one an inward K^+^ current (Matthias et al., 2003). Application of glutamate or AMPA on weak fluorescent GFAP cells evoke a fast and sensitized current whether d-aspartate does not evoke any current, indicating expression of AMPA receptors and lack of glutamate transporters. On the contrary, the second group of cells has no kainate elicited membrane currents, however, d-aspartate induce inward currents inhibited by the use of glutamate transporter blocker THA (Matthias et al., 2003).

Investigation on the existence of astrocytes with qualitatively different ion current phenotypes and morphology in the hippocampus (Zhou and Kimelberg, 2000, 2001) and thalamus (Hoft et al., 2014) suggest astrocyte heterogeneity by diverse functional properties in different populations of cells, some of which expressing functional AMPA receptors and others glutamate transporters (Matthias et al., 2003). Knowing the importance of neurotransmitters for the regulation of many signaling pathways, further studies are relevant in order to reveal the role of the differential expression of glutamatergic machinery in astrocytes to physiological events such as cell migration and differentiation.

A distinct feature of astrocytes is their extensive gap junctional coupling. Gap junction communication is essential for signaling in neuroglia circuit function in many brain regions. Therefore, coupling differences among those regions could indicate astrocyte heterogeneity with possible functional diversity implications (Froes and Menezes, 2002; Anders et al., 2014). Even more subtle paradigms to measure dye diffusion via gap junction reveals differences in astrocyte performance with temperature variance. Experiments using the fluorescent dye Alexa Fluor 594 to measure those parameters in CA1 and dentate gyrus of the rat hippocampus (Anders et al., 2014) show that astrocyte coupling may differ between these regions in a temperature-dependent manner, probably due to changes in intracellular diffusive properties, rather than measured by the number of astrocytes coupled (Anders et al., 2014).

## The Origin of Astrocytes

Gliogenesis generally follows neurogenesis in the developing brain (Miller and Gauthier, 2007; Costa et al., 2009). However, these events partially overlap and their precise temporal relationship *in vivo* and at the individual progenitor level remains largely unexplored. Most macroglial cells in the rodent brain are generated postnatally. In fact, during the first 3 weeks of cerebral cortex postnatal development, the macroglial cell population, which contains predominantly astrocytes, expands six- to eightfold in the rodent brain (Bandeira et al., 2009).

As discussed above, astrocyte diversity in the brain becomes increasingly recognized. Yet, it remains unclear whether astrocyte subtypes are generated from a homogeneous population of progenitors or from separate classes of progenitors previously specified within the germinative niches of the developing telencephalon. Moreover, the developmental sequences undergone by astrocyte precursors are only partially understood. We summarize the main findings related to the generation of differentiated astrocytes in the brain parenchyma.

**FIGURE 1:**
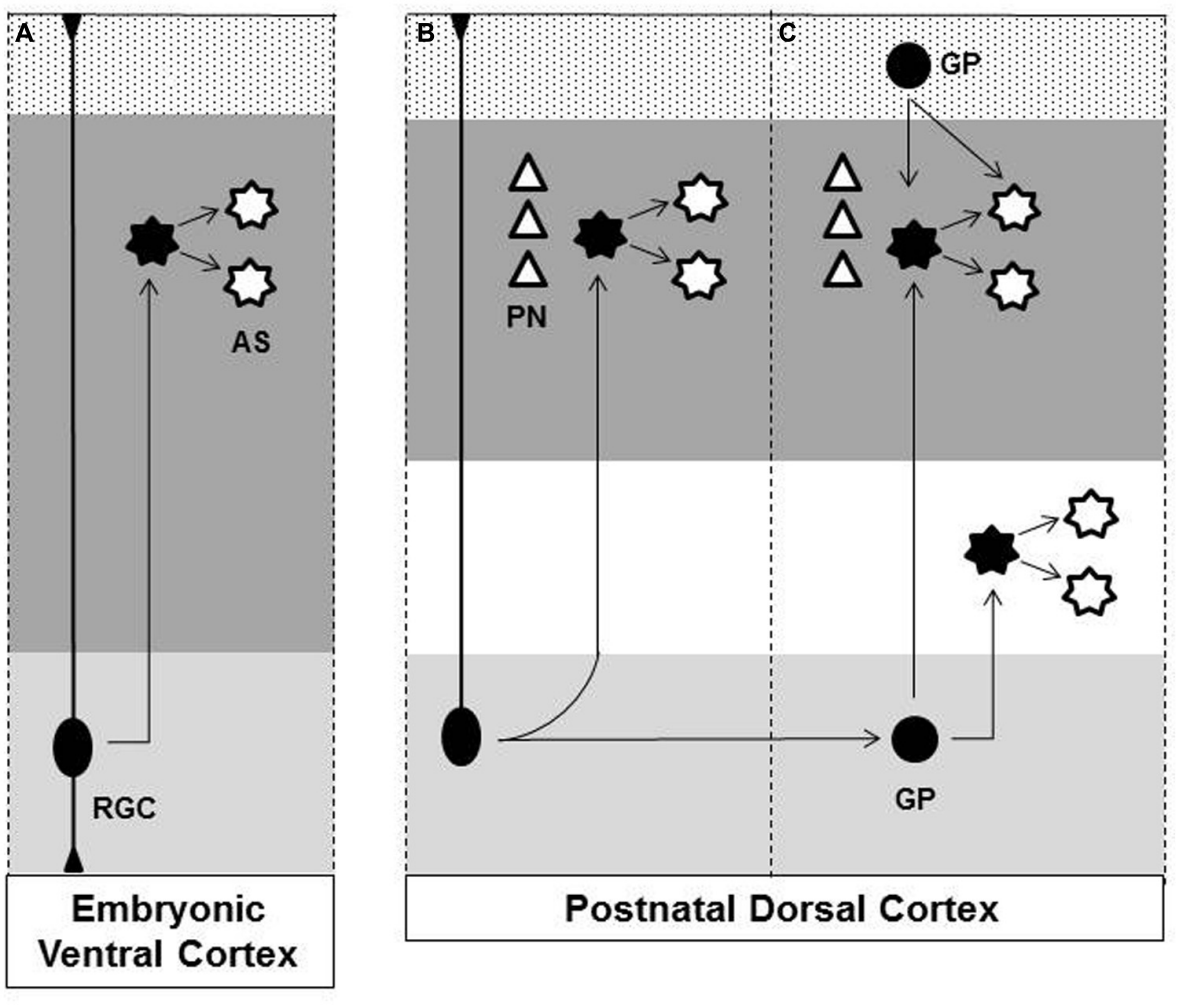
**Origins of astrocytes in the cerebral cortex. (A)** At later stage of embryonic brain development radial glial cell (RGC), in the ventricular zone (light gray region), gives rise to astrocytes (AS) that disperse throughout the ventral forebrain parenchyma (dark gray region). **(B)** After birth, RGC loses their apical processes and directly transform into cortical astrocytes. **(C)** Glial progenitor (GP) derived from RGC undergoes cell division in the subventricular zone (light gray region) generating astrocytes that disperse radially to the cortical layers (dark gray region) and white matter (white region). Astrocyte proliferates locally amplifying the astrocytic population. GP present in the Marginal Zone (MZ)/Layer 1 (Dotted region) contributes to superficial cortical astrocyte. **(B/C)** During embryonic development dorsal RGC also generates pyramidal neurons (PN) that migrate radially and settle in individual cortical columns in the gray matter. Observe that developing astrocytes maintain the columnar organization with early generated neurons. Dashed lines indicate the boundaries of individual cortical columns. Black filled figures represent mitotic cells. RGC, radial glia cell; AS, astrocyte; GP, glial progenitor; PN, pyramidal neurons.

Five different sources of cortical mature astrocytes were identified to date: (I) radial glia cell (RGC) within ventricular zone, (II) RGC transformation, (III) glial intermediate progenitors (GP) within subventricular zone, (IV) GPs present in the marginal zone (MZ)/layer 1, (V) superficial layer progenitors. A schematic view of astrogliosis to the cerebral cortex is illustrated in the **Figure 1**.

### (I, II and III) Astrocytes Derived from Radial Glia Cell Directly, or by Direct Transformation or by the Generation of Intermediate Progenitors

One of the earliest and most understood source of astrocytes in the cerebral cortex is the direct transformation of RGCs into protoplasmic astrocytes that occurs, at the end of cortical neurogenesis, after birth in different species (Schmechel and Rakic, 1979; Voigt, 1989; Alves et al., 2002; deAzevedo et al., 2003). During this process, RGCs lose their apical process and move toward the pial surface, eventually undergoing mitosis in the subventricular zone (SVZ; Noctor et al., 2004). It is likely that these transforming and proliferating cells represent at least a fraction of the GPs labeled by retrovirus in the SVZ and studied in previous work (Levison et al., 1993), what could help to explain the little dispersion of clonally related astrocytes (Magavi et al., 2012).

Radial glia cells generate intermediate progenitors through asymmetric division within the SVZ. These glial intermediate progenitors also divide and give rise to immature proliferative astrocytes that migrate radially out of the germinative zone and populate the cerebral parenchyma (Levison and Goldman, 1993; Levison et al., 1993; Luskin and McDermott, 1994; Zerlin et al., 1995). When they reach their destination, the immature cells still proliferate and only later fully differentiate into mature astrocytes (Ge et al., 2012).

Interestingly, retroviral-mediated fate mapping of postnatal SVZ progenitors indicate that white and gray matter astrocytes, as well as oligodendrocytes derive from separate macroglial progenitors (Luskin and McDermott, 1994) and that astrocytes generated in the SVZ do not disperse long distances in the rostro-caudal axis (Levison et al., 1993), instead they are regionally restricted. This regionalization may start at earlier time points, as indicated by recent work (Magavi et al., 2012; Garcia-Marques and Lopez-Mascaraque, 2013; Gao et al., 2014). Using a novel genetic tool for tracing GP lineage, the so-called “Star Track,” which uses a combinatorial expression of six fluorescent proteins under the control of GFAP promoter, Garcia-Marques and Lopez-Mascaraque (2013) show that clonally related astrocytes disperse radially in the cortex spanning the entire depth of the cortical parenchyma. Similarly, when individual cortical RGCs are labeled at early embryonic stages using genetic strategies, they give rise to discrete columnar structures that contain both projection neurons and protoplasmic astrocytes in the adult animals (Magavi et al., 2012; Gao et al., 2014). Most columns of neurons contained multiple clusters of astrocytes, and the vast majority of labeled astrocytes were found within 50 μm of a labeled neuronal column. The astrocyte to neuron ratio in a single developmental column was similar across the entire neocortex, indicating that column-associated astrocytes account for the majority of protoplasmic astrocytes in the neocortex. Together these data suggest that cortical protoplasmic astrocytes are generated in a spatially restricted manner from progenitors that also give rise to columns of pyramidal neurons (PNs) during embryonic development.

Notably, cortical excitatory neurons are spatially organized into individual cortical columns, which are involved in the processing of similar sensory stimuli (Mountcastle, 1997). It is hypothesized that the generation of neuronal cortical columns could be controlled by transcriptional codes expressed in progenitor cells at early development (Costa and Hedin-Pereira, 2010) and it is tempting to speculate that the columnar organization of astrocytes in the cerebral cortex (Magavi et al., 2012) could also reflect some degree of spatial specification in GPs. Some evidence of this was found in spinal cord where astrocytes have been shown to be regionally specified (Hochstim et al., 2008). In this work, the authors describe three distinct subtypes of astrocytes which could be distinguished by the combinatorial expression of Reelin and Slit1 (Hochstim et al., 2008). These astrocyte subtypes originate from separate progenitor domains expressing the transcription factors Pax6 and Nkx6.1, suggesting that astrocyte diversification could be regulated within progenitors.

Another example of specific progenitors which generate spatially restricted astrocyte subpopulations comes from lineage studies of ventral telencephalon NG2 cells. It is found that ventral embryonic NG2 cells, derived directly from RGCs, generate a subset of protoplasmic astrocytes only to the ventral forebrain, but no astrocytes to the dorsal cortex or cortical white matter, e.g., corpus callosum. (Marshall and Goldman, 2002; Belachew et al., 2003; Zhu et al., 2008, 2012; Huang et al., 2014). Moreover, embryonic NG2 cells only generate astrocytes within the forebrain, since no labeled astrocytes were found in other brain regions (Huang et al., 2014). Thus astrogliogenic potential of NG2 cells seems to be temporal and spatially confined to a subgroup of ventral embryonic progenitors.

On the other hand, dorsal cortical astrocytes are mostly generated from an Emx1-expressing progenitor (Gorski et al., 2002), which is a transcription factor regionally expressed by cortical progenitors in the dorsal VZ/SVZ (Gulisano et al., 1996). Using a conditional double transgenic mouse reporter to trace Emx1-cell lineage, the authors show that the vast majority of, if not all, cortical excitatory neurons and astrocytes generated postnatally is derived from Emx1-expressing cells (Gorski et al., 2002). Thus ventral and dorsal forebrain astrogliogenesis are temporal and spatially confined to a subgroup of GPs. While subpallial astrocytes are dependent on NG2-expressing progenitors, pallial astrocytes rely on Emx1-expressing progenitors.

### (IV) From Glial Progenitors Present in the Marginal Zone/Layer 1

In the embryonic and neonatal MZ/layer 1, a separate class of progenitors undergoes cell division and contributes with astrocytes, oligodendrocytes, and neurons to the cerebral cortex (Costa et al., 2007; Breunig et al., 2012) and therefore is described as a novel niche for gliogenesis and neurogenesis in the cerebral cortex. MZ/layer 1 progenitors are derived from ventral (progenitors expressing the transcription factors Nkx2.1 or Gsh2) and dorsal telencephalic ventricular zone (progenitors expressing the transcription factors Emx1), with a predominance of the latter at neonatal stage (Costa et al., 2007). Given the unique composition of the MZ/layer 1, it is possible that local proliferation in this region contributes to the generation of astrocyte diversification in the cerebral cortex (Costa et al., 2007). Actually, evidence from human embryonic cortex shows that different morphologies and developmental stages characterize superficial and deep astrocytes (deAzevedo et al., 2003). Whilst deep astrocytes emerge by the process of RGC transformation in the subplate of human cingulate cortex, superficial astrocytes labeled for GFAP are already present in the MZ and dorsal supragranular layers in the second half of gestation. At this point no GFAP-positive cells can yet be labeled in human cortical plate. The first GFAP-positive cells to be labeled within the human cortical plate appear associated with blood vessels (Marin-Padilla, 1995; deAzevedo et al., 2003) raising the question of whether angiogenesis might have a pivotal role in the appearance of gray matter astrocytes. While in humans GFAP is a conventional marker for RGCs and astrocytes, in rodents this labeling is restricted, bringing our attention to the evolutionary differences in the glial population between rodents and primates, for example (Takahashi et al., 1990; deAzevedo et al., 2003).

### (V) Superficial Layer Progenitors

Local proliferation was the main focus on a recently study of Ge et al. (2012). They show that GPs and RGC transformation combined contribute to mature astrocyte differently when observed superficial and deeper cortical layers. While both sources generate most of the astrocytes to deep cortical layers (layers 5–6) and white matter astrocytes, 97% of labeled progenitors, only 3% of these progenitors are dedicated to upper cortical layers (layers 1–4). Using retrovirus injected in the cortex of postnatal mice, the authors labeled dividing cells and provide evidence, at least to superficial cortical layers, that local proliferation of immature astrocytes is responsible to expand the cell population. It is possible that local proliferation in deeper cortical layers and white matter may also occur. However, more striking is to determine whether this differential astrocyte dispersion through cortical layers confers a distinct glial function within neuronal circuitries. It is also unclear whether these locally proliferating astrocytes are derived from GPs in the MZ/Layer 1.

Future experiments should address more directly the question of whether positionally and functionally distinct subtypes of astrocytes in the forebrain develop from previously specified sets of progenitors and the degree of plasticity of GPs when proliferating in different environments, such as MZ/Layer 1, deep and superficial cortical layers, or white matter. These studies may also shed light on our understanding about the mechanisms subsidizing neurological and psychiatric diseases in which astrocytes are affected at subpopulation distinct manner.

## Astrocytes and Disease

Given the diversity of astrocytes and their pleiotropic functions, it is not surprising that their dysfunction is also an important matter to several neurological disorders. However, the involvement of this type of glia in pathological conditions became only recently clear due to a steadily increasing interest in the study of the biology and pathology of astrocytes (Sofroniew and Vinters, 2010). It is tempting to speculate that different subtypes of astrocytes would contribute to the onset or progression of brain diseases. And even more, only a subset of astrocytes would be differentially affected by the microenvironment of the diseased brain.

The response of astroglia in pathological conditions is very heterogeneous. Indeed, in some circumstances, it is possible to observe morphological changes of these cells that become hypertrophic and proliferate, leading to the so-called reactive gliosis state. In these conditions, astrocytes can release different kinds of cytokines with either pro-or anti-inflammatory potential (Sofroniew and Vinters, 2010). Other pathological situations are characterized by astro-degeneration with consequent loss of their physiological supportive functions (Scuderi et al., 2013). Nowadays, there is increasing evidence of astrocytic dysfunction in several brain disorders. The homeostatic failure due to astrocyte degeneration can be fundamental for the initiation and progression of neuropathological diseases. There is an increasing body of evidence showing the contribution of astrocytes in schizophrenia (Kolomeets and Uranova, 2010; Schnieder and Dwork, 2011), autism (Laurence and Fatemi, 2005; Bristot Silvestrin et al., 2013), and drug abuse (Beardsley and Hauser, 2014; Bull et al., 2014; Jackson et al., 2014). Astrocyte dysfunction is also involved in major depression disease and neurodegenerative disorders such as Alexander Disease (AxD), Amyotrophic Lateral Sclerosis (ALS), and Alzheimer’s Disease (AD), focused on this review. Although astrocytes present a common reaction to the lesioned nervous system with the upregulation of GFAP for example, we will see in the following session that there is enormous diversity in astrocyte response that may be accounted for by environmental differences or by astrocyte heterogeneity.

### Amyotrophic Lateral Sclerosis

Amyotrophic lateral sclerosis is a neurodegenerative disease that is characterized by the loss of corticospinal and spinal motor neurons. Multiple genes are linked to this disorder, but the discovery of mutations in the Cu-Zn superoxide dismutase (SOD1) led to the generation of transgenic animal models that have contributed to current knowledge on ALS pathology. Evidence support a central role for neuro–glia interactions suggesting that glial cells, and in particular astrocytes, could be a target for novel therapy in ALS (Valori et al., 2014). In fact, massive activation of astrocytes and microglia is associated with motor neuron death in humans, as well as in transgenic animal models (Boillee et al., 2006; Yamanaka et al., 2008). However, selective ablation of proliferating GFAP-expressing astrocytes and microglia fail to modify disease progression (Gowing et al., 2008; Lepore et al., 2008). Using SOD1^G93A^ transgenic (SOD–Tg) rats, Barbeito and collaborators isolated in culture a new type of astrocyte from adult spinal cord of symptomatic animals (Diaz-Amarilla et al., 2011). SOD–Tg-derived astrocytes reached confluence rapidly and could be propagated for 1 year in culture. Because of this feature they were called “aberrant astrocytes” (AbA cells), which are highly proliferative and appear to drive motor neuron death in a cell-type specific fashion *in vitro* (Diaz-Amarilla et al., 2011).

Aberrant astrocytes cells are almost undistinguishable morphologically from primary neonatal astrocytes. They express a set of distinctive antigenic markers of undifferentiated astrocytes including intense staining for S100β in the cell nucleus and cytoplasm, high connexin 43 expression and low levels of diffuse perinuclear labeling of non-filamentous GFAP (Diaz-Amarilla et al., 2011). This low expression of GFAP could be partially responsible for failure observed in earlier studies, which aimed to control proliferation of GFAP-expressing astrocytes in ALS lesion.

Interestingly, AbA cells do not express detectable GLT-1, a specific glial glutamate transporter, what could explain further excitotoxic damage to motor neurons *in vivo*. Moreover, the neurotoxicity of AbA cells conditioned medium is specific to motor neurons (Diaz-Amarilla et al., 2011). This cell-type specific interaction has been already reported for mutant SOD1 expressing primary astrocytes, which reduce viability of both primary and embryonic stem cell-derived motor neurons in co-culture, but not interneurons or dorsal root ganglion neurons (Nagai et al., 2007).

In culture, AbA cells also show increased proliferation and lack of replicative senescence, suggesting there is a defect in contact inhibition, but they do not appear to be fully transformed cells (Diaz-Amarilla et al., 2011). Additionally S100β expression did not co-localize with NG2-labeling cells, which proliferate in the ALS spinal cord (Kang et al., 2010), suggesting that the dividing S100β-positive cells constitute a different cell population that is not derived from NG2 cells. Recently, it was found that AbA cells are most likely derived from activated microglia present in the spinal cord lesion (Trias et al., 2013). After two weeks in culture, SOD-Tg-derived microglia cells start their transformation into astrocytes. Thus, a subpopulation of microglia in culture loses its markers, such as Iba1 and CD163, and increases the expression of the astrocyte markers GFAP and S100β. Changes in the protein profile are also followed by phenotypical alterations (Trias et al., 2013). This finding raises a novel perspective on astrocyte studies in ALS disease, which should then consider impeding the transformation of microglia into astrocyte-like cells as a therapeutic strategy.

For all these evidence, authors suggest that AbA cells may be considered a distinct subpopulation of highly toxic astrocytes generated during recruitment and phenotypic transition of glial cells in an inflammatory microenvironment. And since astrocytes are involved in the rapid progression of paralysis characteristic of the ALS animal model, AbA cells could represent an additional cellular target for future treatment of ALS (Diaz-Amarilla et al., 2011; Trias et al., 2013).

### Alexander Disease

Alexander disease is an autosomal dominant leukodystrophy, which predominantly affects infants and children (Goyal et al., 2014; Verkhratsky and Parpura, 2014). It is an inherited gliopathology, associated with sporadic mutations in the GFAP encoding gene that was first described in 1949 by Stewart Alexander (Alexander, 1949; Brenner et al., 2001; Verkhratsky and Parpura, 2014). Those pathogenic mutations are thought to confer cytotoxicity through gain-of-function mechanisms (Brenner et al., 2001; Prust et al., 2011). The impaired function of astroglia affects brain development and results in progressive failure of central myelination, developmental delay, seizures, megalencephaly, and progressive deterioration, with increased severity in neonatal patients (Prust et al., 2011; Verkhratsky and Parpura, 2014). Histopathological analysis shows that AxD is associated with the appearance of cytoplasmic inclusions in astroglial cells known as the Rosenthal fibers (Verkhratsky and Parpura, 2014). These are protein aggregates containing GFAP, ubiquitin, heat shock protein hsp-27, and B-crystallin and they are expressed in astrocytes adjacent to areas of demyelination (Goyal et al., 2014).

Observation that Rosenthal fiber formation can be induced by the overexpression of human GFAP in transgenic mice in a dose dependent manner (Messing et al., 1998; Quinlan et al., 2007) lead to the search for mutations in the GFAP gene. *Gfap* encodes for an intermediate filament protein that can be alternatively spliced (Quinlan et al., 2007). Relative abundance of GFAP transcripts is often low, dependent on astrocyte location, and induced by disorders (Roelofs et al., 2005; Kamphuis et al., 2012, 2014), suggesting an interesting source for astrocyte heterogeneity. Brenner et al. (2001) found that the genetic mechanism of AxD is based in the *de novo* mutations in four different GFAP residues, R79, R239, R258, and R416 observed in 12 unrelated individuals. In addition, other investigators found several spots for mutations in GFAP gene in early and later onset, indicating dominant missense GFAP mutations for nearly all forms of this disorder (Li et al., 2005; Quinlan et al., 2007). The genetic basis for AxD is now very well established, however, little is known about the mechanisms by which GFAP mutations lead to disease. To understand how the pathology progresses, transgenic mice overexpressing wild type GFAP that develop an encephalopathy with identical aggregates present in AxD were analyzed in different phases by microarray assays (Hagemann et al., 2005; Quinlan et al., 2007). Transcription profiles reveals alteration in genes involved in stress and immune responses (Hagemann et al., 2005). At 3 months age, transgenic mice show stress responses including increase in genes expression involved in glutathione metabolism, peroxide detoxification, and iron homeostasis (Hagemann et al., 2005). GFAP overexpression in those mice also induces an increase in activation of cytokine, cytokine receptor genes, and complement components. These transcripts are further elevated with age, with additional induction of macrophage-specific markers, suggesting activation of microglia (Hagemann et al., 2005; Quinlan et al., 2007). At 4 months, in contrast to those genes showing increased expression at 3 months, there is a decrease in expression of microtubule-associated proteins (Hagemann et al., 2005). Numerous genes involved in neurotransmission and vesicular transport are also downregulated including both GABA and glutamate receptors (Hagemann et al., 2005; Quinlan et al., 2007). The transcriptional profiles from olfactory bulb also show a decrease in transcriptional factors and signaling molecules involved in neurogenesis such as Dlx family genes (Hagemann et al., 2005). Therefore, this completely remodeled scenario affects neuron-glia signaling, leading to neuronal dysfunction in advanced stages of pathology.

### Alzheimer’s Disease

Neurodegenerative disorders such as AD are the most common diseases of modern society. The gradual and irreversible disturbances in homeostasis, leading to synaptic dysfunction and cognition impairment are characteristic features of the disease. Symptomatically, AD is characterized by marked deficiencies in episodic memory, attention, perception, reasoning, and language as well as altered mood (Mesulam, 1999; Hancock et al., 2014). Pathologically, it is defined by the accumulation of intracellular neurofibrillary tangles, comprised of abnormally phosphorylated tau protein and extracellular plaques, including misfolded forms of the amyloid-β (Aβ) peptide within the brain (Mesulam, 1999; Hancock et al., 2014).

The overall assumption in AD is that astrocyte response involves a generalized overexpression of GFAP and an increase in proliferation. However, a growing body of evidence shows that there are two different populations of astrocytes in AD, revealing the heterogeneity in cell response upon different stimuli and environment.

Studies from a triple transgenic mouse model of AD, which mimics the progression of the disease in humans, show that the patterns of GFAP expression differ among brain areas and during the different phases of the neurodegenerative process (Olabarria et al., 2010; Yeh et al., 2011). The number of GFAP-positive astrocytes does not change with the age of the transgenic animals, however, in mice from 6, 12, and 18 months there is a reduction in volume and area of GFAP-expressing cells in the dentate gyrus, indicating astrocyte atrophy (Olabarria et al., 2010). However, in Aβ plaque surrounding areas, there is an opposite profile of GFAP expression, observed by an increase in GFAP volume and superficial expression pattern in the dentate gyrus and CA1 regions, demonstrating a hypertrophy of astrocytes (Olabarria et al., 2010). Therefore, two different populations of astrocytes are revealed in AD. This concomitant astrocyte atrophy and astrogliosis in AD does not seem to occur in all brain regions. Analyses of the entorhinal cortex, a fundamental structure for cognitive and memory processes, show that in the triple transgenic mouse model of AD, there is a reduction in primary and secondary branches accompanied by a decrease in volume and area of GFAP expression. These morphological changes are observed in 1 month old animals and persist up to 12 months (Yeh et al., 2011). The progression of the disease established by the accumulation of Aβ deposits does not trigger a reactive gliosis, indicating an absence of astrocytic hypertrophy during AD in the entorhinal cortex (Yeh et al., 2011).

Another brain area involved in the symptomatology of AD is the medial prefrontal cortex, which is associated with cognitive, memory, and emotional processes. In this brain area, the number of GFAP-positive cells does not change significantly with age as compared to wild type mice (Kulijewicz-Nawrot et al., 2012). However, at 3 months, transgenic mice show astroglial cytoskeletal atrophy that remains throughout the disease progression (Kulijewicz-Nawrot et al., 2012). Reduction in volume and area of GFAP-positive profiles in the medial prefrontal cortex show a clear layer-specific pattern, with layers 1–2 being strongly affected and similar changes being found in the deep layers 4 and 5, while layer 3 is only affected from intermediary phases of the disease progression (Kulijewicz-Nawrot et al., 2012). In contrast to other brain regions affected in AD, such as the hippocampus (Olabarria et al., 2010; Yeh et al., 2011), no plaque formation is observed in medial prefrontal cortex (Kulijewicz-Nawrot et al., 2012). However, the Aβ aggregates are present, especially in the deeper layers. Those findings show that astrocytic atrophy occurs in early stages of the disease in specific brain areas. This alteration of astrocytes may represent a very relevant aspect for the progression of the disease. Astrocytic dysfunction compromises brain homoeostasis on many levels, reducing brain energy and neurotransmitter homoeostasis, increasing excitotoxicity. In addition, atrophied astrocytes can reduce synaptic coverage, leading to a decrease in number and functional synapses, decreased connectivity, imbalanced neurotransmission, synaptic strength, and synaptic maintenance. These data indicate that astrocytes within distinct brain regions may respond in a very peculiar manner to similar stimuli, supporting the view that astrocytes are heterogeneous and play different roles in disease progression.

At later stages of the AD, the astrocytic morphology is complex. There is formation of senile plaques resulting in astrogliosis revealed by astrocytic hypertrophy, thicker processes, increased volume and area of GFAP-positive profiles surrounding Aβ deposits (Olabarria et al., 2010).

Neuropathology data using human brains show that astrocytes activated by Aβ (Scuderi et al., 2013) secrete pro-inflammatory signals and toxic cytokines that lead to further damage, increasing nitric oxide radicals and TNF-α levels, which in turn triggers a neurodegenerative cascade (Zhang et al., 2010). The inflammation itself can lead to neuronal dysfunction, independently of cell death. The parallel pro-inflammatory cytokine network induces dysfunction in astrocytes in their effort to maintain environment homeostasis, which in turn increases neuronal vulnerability. Thus, astrocyte impairment can occur during early and late stages of the disease depending on the brain region and how astrocytes modulate GFAP expression and the secretion of cytokines or trophic factors in response to stimuli.

Glial fibrillary acidic protein gene can be alternatively spliced and the canonical isoform GFAP-α expressed in astrocytes contains nine exons (Kamphuis et al., 2012, 2014). So far, nine isoforms are described in different species (Kamphuis et al., 2012). Three splice variants GFAPDEx6, GFAPD164, and GFAPD lacking sequences in exons 6–7 are found in AD (Hol et al., 2003). GFAP transcripts from alternative splicing have variable alterations in their C-terminal region. The C-terminal region is important to direct the assembly of GFAP filaments and their interaction to other proteins (Kamphuis et al., 2014). Thus, different C-terminals lead to different GFAP expression patterns and cellular functions. Evidence that GFAP can be translated from different transcripts corroborates the idea that astrocytes are distinct cells populations with a specific transcriptional regulation repertoire leading to putative differences in their function.

### Major Depression Disorder

Major depressive disorder (MDD) is one of the most prevalent mood disorders, affecting millions of people worldwide. MDD is a chronic, recurrent and debilitating mental illness, characterized by core symptoms such as depressed mood, loss of interest or pleasure, changes in weight and in sleep, fatigue or loss of energy, feeling of worthlessness, concentration difficulties, and thoughts of death or suicide (Rajkowska and Stockmeier, 2013). Several hypotheses including chronic stress, failure of hippocampal neurogenesis in the adult, altered neuroplasticity, dysfunction of monoaminergic systems and genetic factors have been studied to elucidate depressive-related behaviors (Smialowska et al., 2013). The general knowledge about depression was originally taken from studies showing impairment in the monoamine system and is supported by the understanding of both the pathophysiology of depression and the action of pharmacological treatments. However, in the past few years, this concept related to monoamines has shifted to a putative deficit in glutamatergic signaling contributing to depressive symptoms (Catena-Dell’Osso et al., 2013; Smialowska et al., 2013). Indeed, the use of ketamine, a *N*-methyl-d-aspartate (NMDA) receptor antagonist, provided the most promising results in preclinical studies and produced a consistent and rapid, although transient, antidepressant effect with a good tolerability profile in humans (Catena-Dell’Osso et al., 2013).

Astrocytes play a remarkable range of roles to maintain homeostasis and optimum neuronal function. As mentioned before, astrocytes can remove the excess of K^+^ and water, reuptake and release neurotransmitters, secrete trophic factors, and regulate metabolic pathways. Astroglial cells are the most important cells in the balance of glutamate and GABA signaling due to their ability to uptake those transmitters, control their release and to provide glutamine for glutamate and GABA synthesis. Therefore, astroglial homeostatic cascades are neuroprotective and can prevent neuronal damage by maintaining brain metabolism and attenuating excitotoxicity through removal of glutamate excess (Verkhratsky et al., 2014).

Histopathological studies from *postmortem* brain tissue reveal prominent glial pathology in MDD. Astroglial changes are represented by a decrease in density of astrocytes stained by Nissl technique as well as a decrease in the number of GFAP-positive astrocytes (Rajkowska and Stockmeier, 2013). These alterations in astrocyte number and morphology are observed in many brain regions from MDD subjects. Cortical layers from prefrontal cortex (Cotter et al., 2002), orbitofrontal cortex, subgenual cortex (Ongur et al., 1998), anterior cingulate cortex (Cotter et al., 2001) the hippocampus, and the amygdala (Ongur et al., 1998; Bowley et al., 2002; Rajkowska and Stockmeier, 2013; Verkhratsky et al., 2014) were analyzed with different methodologies and display glia reduction and or morphology alteration (Verkhratsky et al., 2014). Increase in the levels of S100β in blood serum of MDD patients, which is attenuated by antidepressant treatment is another evidence of astrocyte degeneration in the disease (Schroeter et al., 2002; Smialowska et al., 2013).

In contrast to these data, MDD elderly subjects show an increase in GFAP density in cortical layers 3, 4, and 5 of dorsal prefrontal cortex compared to younger MDD patients (Miguel-Hidalgo et al., 2000; Verkhratsky et al., 2014). Indeed, cingulate cortex and orbitofrontal from MDD elderly patients did not show any reductions in GFAP expression (Khundakar et al., 2011a,b; Rajkowska and Stockmeier, 2013), indicating a possible difference in GFAP expression upon aging. There is some evidence that do not confirm GFAP reduction in MDD. Results from entorhinal cortex show no alterations in density and GFAP morphology in MDD subjects (Damadzic et al., 2001). Torres-Platas et al. (2011) demonstrates a hypertrophy in white matter astrocytes in studies from post mortem samples of anterior cingulate cortex of suicide subjects. This observation corroborates data showing inflammatory processes underlying MDD pathology (Maes et al., 2009; Rajkowska and Stockmeier, 2013). In general, apart from data associated with aging or inflammation, there is a decrease in GFAP expression and other related glial markers in brain areas associated with mood disorders (Smialowska et al., 2013).

Reduction of the number of GFAP-positive cells in MDD patients is accompanied by a reduction in the expression of several genes involved with glutamate signaling, mainly expressed in astrocytes in the locus coeruleus (Bernard et al., 2011). *In situ* hybridization data from animal models of MDD also show a reduction in the expression of the GLT-1 in the hippocampus and cerebral cortex (Zink et al., 2010), suggesting a dysfunction in glutamate reuptake, glutamine synthesis and in the glutamate-GABA shunt, possibly underlying the pathology of MDD (Rajkowska and Stockmeier, 2013; Verkhratsky et al., 2014).

Expression of aquaporin 4 and connexins (Cx30 and Cx43) is also reduced in cortical and subcortical astrocytes in both MDD and in an experimental stress model (Rajkowska and Stockmeier, 2013; Verkhratsky et al., 2014). Accordingly, work by Sun et al. (2012) shows that animals submitted to unpredictable stress, a rodent model of depression, exhibited significant decrease in diffusion of gap junction channel permeable dye and expression of Cx43. Furthermore, injections of carbenoxolone, a blocker of gap junctions, into the prelimbic cortex induce anhedonia and anxiety in mice submitted to different behavioral tests (Sun et al., 2012). These data suggest that alteration in mice behavior related to MDD pathophysiology may involve astrocytic communication failure, at least in part, since CBX is an unspecific blocker. This altered scenario can be crucial for the information processing and the establishment of MDD pathophysiology (Verkhratsky et al., 2014).

## Conclusion

Taken together, many of the cellular and molecular markers for astrocyte heterogeneity were shown to be key players in astrocyte mediated disease processes. However, the precise role of different astrocyte populations in disease onset and progression still remains to be addressed.

## Conflict of Interest Statement

The authors declare that the research was conducted in the absence of any commercial or financial relationships that could be construed as a potential conflict of interest.
